# Suppression of experimental autoimmune encephalomyelitis by ultraviolet light is not mediated by isomerization of urocanic acid

**DOI:** 10.1186/s12868-016-0323-2

**Published:** 2017-01-05

**Authors:** Amy A. Irving, Steven J. Marling, Lori A. Plum, Hector F. DeLuca

**Affiliations:** Department of Biochemistry, University of Wisconsin-Madison, 433 Babcock Drive, Madison, WI 53706 USA

## Abstract

**Background:**

Ultraviolet B irradiation confers strong resistance against experimental autoimmune encephalomyelitis, a model of multiple sclerosis. This protection by ultraviolet B is independent of vitamin D production but causes isomerization of urocanic acid, a naturally occurring immunosuppressant.

**Methods:**

To determine whether UCA isomerization from *trans* to *cis* is responsible for the protection against experimental autoimmune encephalomyelitis afforded by ultraviolet B, *trans*- or *cis*-urocanic acid was administered to animals and their disease progression was monitored.

**Results:**

Disease incidence was reduced by 74% in animals exposed to ultraviolet B, and skin *cis*-urocanic acid levels increased greater than 30%. However, increasing skin *cis*-urocanic acid levels independent of ultraviolet B was unable to alter disease onset or progression.

**Conclusions:**

It is unlikely that urocanic acid isomerization is responsible for the ultraviolet B-mediated suppression of experimental autoimmune encephalomyelitis. Additional work is needed to investigate alternative mechanisms by which UVB suppresses disease.

## Background

Multiple sclerosis (MS), a demyelinating disease of the central nervous system, is less prevalent in locations that receive greater amounts of sunlight. This observation has led to the hypothesis that vitamin D might play a major role in preventing the disease. However, despite a correlation between low serum 25-hydroxyvitamin D (25-OH-D) levels and higher disease incidence in human populations, evidence that vitamin D can reduce the incidence of MS has so far not been obtained [1–3]. In experimental autoimmune encephalomyelitis (EAE), a model for MS, 1,25-dihydroxyvitamin D_3_ (1,25-(OH)_2_D_3_) does reduce disease, but only when accompanied by elevated serum calcium [4, 5]. Strikingly, mice deficient in vitamin D or lacking the vitamin D receptor have each been shown to be resistant to developing EAE [6, 7].

MS is an autoimmune disease in which activated T cells target and cause the destruction of the myelin sheath in the central nervous system. In addition to its role in generating vitamin D in the skin, ultraviolet light can cause systemic suppression of the immune system [8]. Ultraviolet B (UVB) irradiation, especially the narrow band from 300 to 315 nm, confers resistance against EAE [9]. This narrow band is exclusive of that required to generate vitamin D in the skin. UV light must then confer protection by mechanisms distinct from vitamin D.

One candidate for this protection is the chromophore urocanic acid (UCA), an intermediate in the catabolism of l-histidine [10]. Under normal conditions, urocanic acid accounts for up to 0.5% of the dry weight of the epidermis, the majority of which is the *trans* isomer [11]. Upon UV exposure of the epidermis, *trans*-UCA isomerizes to the *cis* isomer, which possesses immunosuppressive properties. The greatest degree of isomerization occurs within the same window demonstrated to offer protection from EAE (300–310 nm) [12]. Further, MS patients have significantly lower blood levels of *cis*-UCA compared to controls [13].

In the current study, we tested *trans*- and *cis*-UCA, as well as *trans*-UCA plus UVB for potential to prevent the onset or progression of EAE.

## Methods

### Animals and diet

Female C57BL/6 mice were purchased from Jackson Laboratory at 8–9 weeks of age and allowed to acclimate to the facility for ~1 week before experimental manipulations began. Mice were maintained in the Department of Biochemistry vivarium with a 12 h:12 h light:dark cycle and fed a standard lab diet chow 5008 (Purina Mills, Richmond, IN). All procedures were approved by the Research Animal Resources Committee of the College of Agricultural and Life Sciences at the University of Wisconsin-Madison.

### EAE induction and scoring

**Fig. 1:**
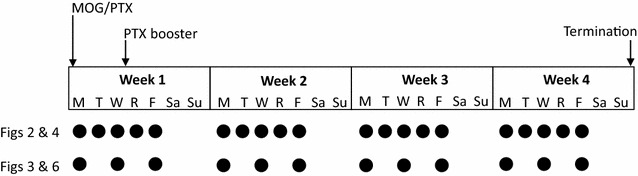
Schematic of the experimental timeline for each experiment. The days of the week are denoted by abbreviations below each of the week designations. The immunization schedule and termination are shown above the timeline. Each *closed circle* represents an experimental exposure (to either UVB, compound or the combination). The Figures listed to the left of the *circles* are those which correspond to that particular experimental timeline. *MOG* myelin oligodendrocyte glycoprotein; *PTX* pertussis toxin

Mice were immunized at 9–10 weeks of age with myelin oligodendrocyte glycoprotein peptide (MOG)_35–55_ purchased from Hooke Laboratories (Lawrence, MA). MOG_35–55_ (MEVGWYRSPFSRVVHLYRNGK) is emulsified in complete Freund’s adjuvant and contains inactivated *Mycobacterium tuberculosis* H37Ra. On day 1, each mouse was injected subcutaneously with 10 μl of MOG emulsion and injected intraperitoneally with 200 ng of pertussis toxin (List Biological Laboratories, Campbell, CA) diluted in sterile PBS. On day 3, a second 200 ng booster pertussis toxin injection was given (Fig. 1). Each mouse was scored daily Monday through Friday for clinical signs of EAE using the following scale: 0, no clinical disease; 1, loss of tail tone; 2, unsteady gait; 3, hind limb paralysis; 4, forelimb paralysis; 5, death.

### UVB radiation treatment

The dorsal surface of each mouse was shaven using an electric razor prior to treatment. Mice were irradiated with a bank of UVB lamps that emit from 300 to 315 nm with a peak at 311 nm (Amjo Corp, West Chester, OH). The radiation output was measured by placing a UV radiometer equipped with 302 and 365 nm sensors (UVP LLC, Upland, CA) at five locations within the cage, representing the positions occupied by the mice. An average output was calculated and the time adjusted to expose the mice to 10 kJ/m^2^ per treatment. These readings were confirmed using a wide band spectroradiometer RPS900 (International Light, Peabody, MA). Mice were irradiated daily Monday through Friday for four weeks (Fig. 1).

### Chemicals

*Cis*-urocanic acid (U6883) was purchased from Sigma-Aldrich (St. Louis, MO) and *trans*-urocanic acid (I0002) was purchased from TCI America (Portland, OR). Both compounds had been determined by the manufacturer to be greater than 98% pure by HPLC.

For topical dosing studies, the compounds were prepared by diluting commercially available *trans*- or *cis*-UCA at a concentration of 1 mg/ml in ethanol (to facilitate rapid skin absorption). Each mouse was then given 2 × 0.2 ml (400 μg total) of either *trans*- or *cis*-UCA onto shaved dorsal skin on Monday, Wednesday and Friday each week during the study (Fig. 1). For the study involving dosing with *trans*-UCA followed by exposure of the mouse to UV, dosing and exposure occurred daily Monday through Friday each week (Fig. 1). Formulations were prepared weekly and concentrations confirmed by HPLC. UCA diluted in ethanol and stored at 4 **°**C is stable for at least 3 months.

For the i.p. dosing study, *trans*-UCA was prepared as above but diluted in sterile PBS. Mixed UCA isomers were generated by exposing 1 mg/ml *trans*-UCA to narrow band UVB (300-315 nm) for 30 min (13.5 kJ). The percent conversion to *cis*-UCA was then measured by HPLC and the absolute concentration of *trans*- and *cis*-UCA determined using quantitative standards. Each mouse was given 0.2 ml (200 μg) of either *trans*-UCA or mixed UCA isomers via i.p. injection on Monday, Wednesday and Friday each week during the study (Fig. 1). Formulations were prepared weekly and concentrations confirmed by HPLC. UCA diluted in PBS and stored at 4 **°**C is stable for at least 6 months.

### Sample preparation

At termination, mice were euthanized with CO_2_ and skin was collected from the dorsal surface, cut into small pieces and frozen at −80 **°**C. A modified Bligh-Dyer method was used to isolate water soluble components, which includes UCA. Skin samples were homogenized on ice in cold water, followed by the addition of methanol/dichloromethane, dichloromethane, and finally water. Samples were then centrifuged for 15 min at 3500 rpm at 4 **°**C. After centrifugation, the water phase was collected, filtered first through a 0.45 μm filter and then again through a 0.22 μm filter, dried down under nitrogen and resuspended in HPLC mobile phase (water containing 0.1% trifluoroacetic acid (TFA)).

### HPLC methods

HPLC separation was performed on a Waters system, composed of a 717plus Autosampler, 600 Controller and Pump, and 996 Photodiode Array Detector (PDA). Empower Pro V5.0 software was used for data acquisition and to control the HPLC system. A Waters Symmetry C18 column (3.9 × 150 mm, 5 μm) maintained at 30 **°**C was used for separation. A gradient from 100% water to 70:30 water:ACN was run over a 15 min period at a flow rate of 0.5 mL/min. Mobile phase contained 0.1% TFA. Data were collected at 264 nm. Retention time for *trans*-UCA was 10.3 min and *cis*-UCA was 14.2 min.

### Statistical analysis

Data are expressed as Mean ± SD. Onset was calculated by averaging the first day when clinical signs appeared. Unless otherwise noted, average clinical scores were calculated at termination on day 28. Mean severity was determined by averaging all clinical scores within a treatment group for a particular day. Statistical calculations indicate that 8 mice per group are required to detect an 80% reduction in average clinical score with 90% power at a significance level of 0.05, as is seen with UVB exposure. If any urocanic acid intervention showed half the efficacy as UVB exposure (~40% reduction in average clinical score), 9 mice per group would give 90% power at a significance level of 0.05. Based on these calculations we have employed 10–12 mice per group to ensure >90% power to detect a statistical difference, if one exists.

Statistical analysis was performed using the two-tailed Fisher exact probability test for incidence, the Wilcoxon rank sum test for clinical scores, and the Kendall rank test for correlation between disease indices and UCA content. A value of P < 0.05 was considered statistically significant.

## Results

### Conversion of urocanic acid by narrow band UVB

Narrow band (NB) UVB was efficient at converting *trans*-UCA to the *cis* isomer both in vitro in solution and in vivo in skin. *In vitro,* starting material was confirmed to be greater than 98% pure *trans* isomer by HPLC. Approximately 13.5 kJ of NB UVB was needed to convert half of the *trans* to the *cis* isomer.

**Fig. 2:**
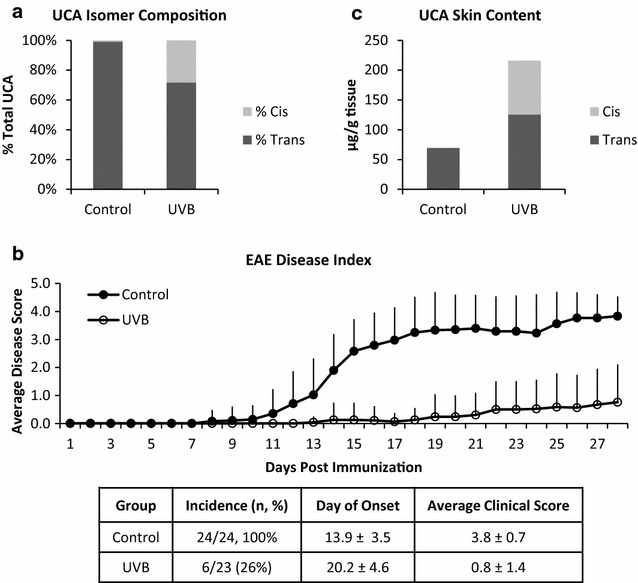
UVB increases *cis*-UCA skin content and efficiently suppresses EAE. **a** UVB significantly increased skin *cis*-UCA content (P < 0.0001) through conversion of the *trans* isomer (**b**). **c** UVB suppresses the onset (P = 0.006) and progression of EAE (P < 0.0001). Data are cumulative of two independent experiments

*In vivo*, under normal conditions, less than 1% of skin UCA content was in the *cis* form. After 4 weeks of UVB exposure, as much as 35% of total skin UCA was the *cis* isomer (Fig. 2a). UVB treatment resulted in a significant suppression of EAE (Fig. 2b). While 100% (24/24) of animals immunized with MOG developed EAE with an average onset of 13.9 ± 3.5 days, UVB reduced incidence to 26% (6/23, P < 0.0001) and delayed onset to 20.2 ± 4.6 days (P = 0.006). UVB also reduced mean clinical score at study termination to 0.8 ± 1.4, compared to 3.8 ± 0.7 in control animals (P < 0.00001). While UVB exposure tripled the total UCA skin content (Fig. 2c), there was no correlation between total or isoform-specific UCA content and day of onset or terminal disease score (data not shown).

### Topical urocanic acid does not prevent EAE

**Fig. 3:**
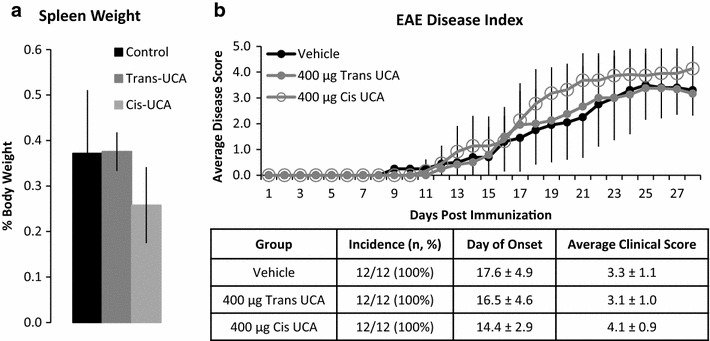
Topical UCA is unable to suppress EAE. **a** *Cis*-UCA significantly decreased the spleen:body weight ratio, while *trans*-UCA had no effect. *Error bars* are SD from the Mean and represent biological variation. **b** Neither UCA isomer was able to suppress EAE onset or progression. Data are representative of one experiment

Topical *trans*- or *cis*-UCA was applied daily to shaven dorsal skin of EAE-induced mice. Indicative of its immunosuppressive properties, topical *cis* decreased average spleen weight 30% compared to vehicle treated animals (P < 0.008, Fig. 3a). Despite this, neither isomer protected against EAE development (Fig. 3b), with an incidence of 100% in each of the groups. Mean onset did not differ from vehicle (17.6 ± 4.9 days) for animals given either *trans* (16.5 ± 4.6 days, P = 0.4) or *cis* (14.4 ± 2.9 days, P = 0.1) compound. Mean clinical score at study termination also did not differ significantly between groups: vehicle 3.3 ± 1.1, *trans* 3.1 ± 1.0 (P = 1 versus vehicle), *cis* 4.1 ± 0.9 (P = 0.07 versus vehicle).

**Fig. 4:**
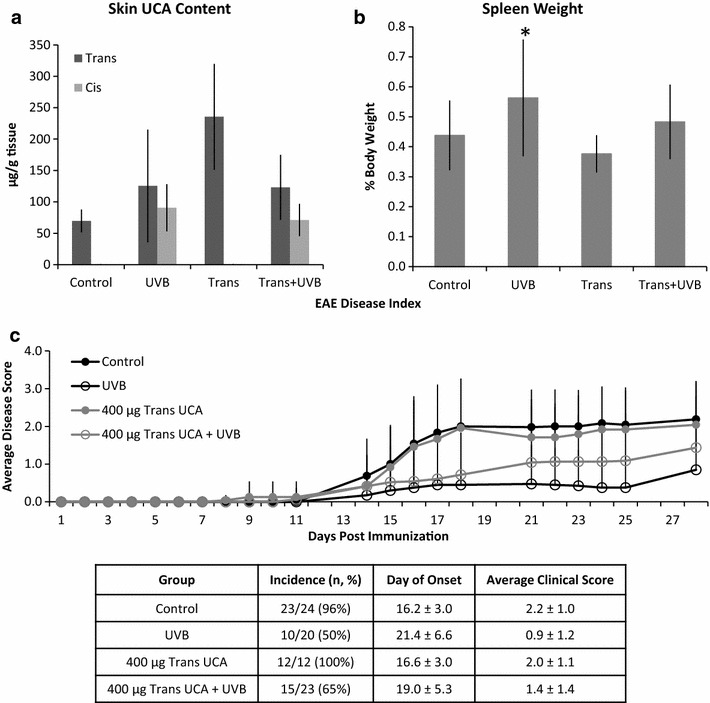
*Trans*-UCA applied topically in combination with UVB is not as effective as UVB alone to suppress EAE. **a** Topical *trans*-UCA significantly increases skin *trans* content. Despite this, when combined with UVB, supplemental *trans* is converted to *cis* to levels similar to that in animals exposed to UVB alone. **b** UVB alone is the only group in which spleen:body weight ratio was significantly increased (P < 0.03). *Error bars* are SD from the Mean and represent biological variation. **c** *Trans*-UCA alone does not affect disease course. UVB greatly suppresses EAE onset and progression. When supplemental *trans*-UCA is given topically prior to UVB exposure, the disease suppression is dampened. Data are cumulative of two independent experiments

A second set of experiments was conducted to determine whether increasing skin *trans* content topically and then exposing that skin to UVB could increase conversion to the *cis* isoform and offer the same or greater protection than UVB alone. While topical application of *trans*-UCA did increase skin *trans* content (P = 0.03, Fig. 4a), spleen weight was unaffected (P = 0.2, Fig. 4b) and disease incidence (12/12) did not differ from vehicle (23/24, P = 1). The greatest disease suppression occurred with UVB exposure alone, where spleen weight was significantly increased (P < 0.03) and incidence significantly decreased (10/20, P < 0.001) compared to vehicle (Fig. 4c). When animals given the *trans* isomer were then exposed to UVB, spleen weight was unaffected compared to vehicle (P = 0.2, Fig. 4b) with an incidence of 65% (15/23, P = 0.01 versus vehicle, P = 0.4 versus UVB alone) (Fig. 4c).

**Fig. 5:**
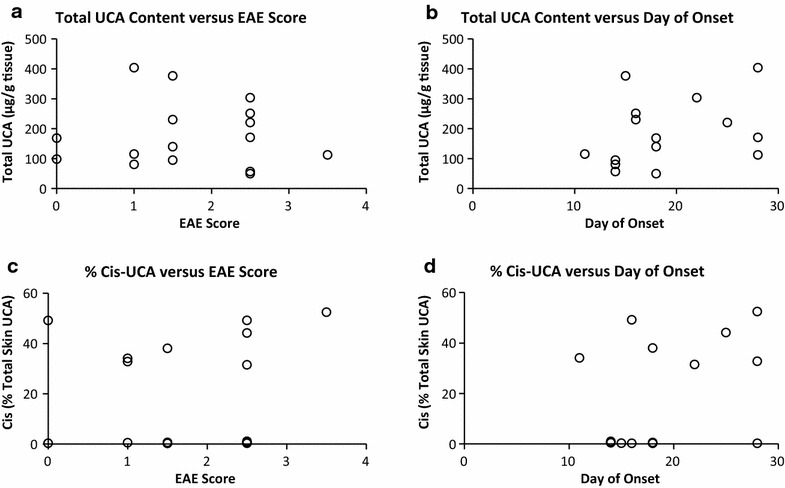
UCA content does not correlate with EAE disease index. There is no correlation between total UCA content (**a**–**b**) nor % UCA in the *cis* isomer (**c**–**d**), and terminal EAE score (**a**, **c**) or day of onset (**b**, **d**)

Interestingly, animals given supplemental *trans* topically and then exposed to UVB were able to convert to the *cis* isoform with similar efficiency to that of animals only exposed to UVB with no additional UCA. However, UVB alone was the only treatment able to significantly delay onset of disease (21.4 ± 6.6 days) compared to vehicle (16.2 ± 3.0 days, P < 0.03). Mean clinical score at study termination only differed from vehicle (2.2 ± 1.0) for animals in the UVB group (0.9 ± 1.2, P < 0.0008), and did not differ for either the *trans* (2.0 ± 1.1, P = 0.8) or *trans* plus UVB groups (1.4 ± 1.4, P = 0.2). Further, there was no correlation between terminal disease score or day of onset and total skin UCA content or % total skin UCA in the *cis* form (Fig. 5), nor absolute *trans* or *cis* skin UCA content (data not shown).

### Systemic delivery of urocanic acid isomers does not prevent EAE

**Fig. 6:**
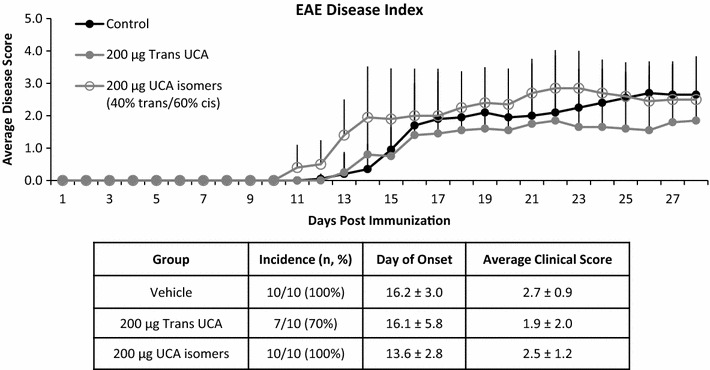
Systemic delivery of UCA via i.p. injection does not prevent development of EAE. Neither day of onset nor disease progression is significantly altered by either *trans*- or mixed UCA isomers. Data are representative of one experiment

To test whether systemic delivery of a mixture of UCA isomers similar to that produced in skin upon UVB exposure could protect against EAE development, mice were injected i.p. three times per week for four weeks with 200 μg of either *trans*-UCA or mixed UCA isomers containing 40% *trans* and 60% cis (generated by exposing a solution of *trans*-UCA to UVB). Neither compound improved disease outcome compared to vehicle (Fig. 6). Incidence was high in all groups: 100% (10/10) for vehicle, 70% (7/10) for *trans* (P = 0.2 versus vehicle), and 100% (10/10) for the mixed UCA isomers. Mean day of onset did not differ significantly between groups: 16.2 ± 3.0 days for vehicle, 16.1 ± 5.8 days for *trans* (P = 0.3 versus vehicle), and 13.6 ± 2.8 days for mixed UCA isomers (P = 0.06 versus vehicle). There was also no difference in mean disease score at termination from control (2.7 ± 0.9) for either the *trans* (1.9 ± 2.0, P = 0.4) or mixed UCA isomer (2.5 ± 1.2, P = 0.6) groups.

## Discussion

MS is not only more prevalent with increasing distance from the equator, but deaths from the disease [14], as well as relapse [15], are also more common. This led to the hypothesis that the vitamin D produced by UVB acting on 7-dihydrocholesterol was responsible for the lowered incidence of MS. However, our group has shown that the vitamin D receptor (VDR) is required for EAE initiation [6], and several groups have demonstrated that animals deficient for vitamin D are protected against EAE [6, 7, 16]. Further, administration of the active form of vitamin D, i.e. 1,25-(OH)_2_D_3_, can suppress EAE in mice but only when hypercalcemia results [5]. Hypercalcemia produced by parathyroid hormone also suppresses EAE [4]. Thus, the idea that vitamin D mediates the suppression of MS by UVB is no longer viable.

As an alternative hypothesis, isomerization of urocanic acid was an attractive candidate to explain the suppression by UVB. Peak isomerization occurs in the UV range most able to suppress EAE [12], the immunosuppressive properties of the *cis* isomer has been demonstrated in multiple animal models [17–19], and an association between low *cis*-UCA levels and MS in patients has been shown [13]. However, we have demonstrated that increasing skin *cis*-UCA levels independent of UVB exposure is unable to protect against EAE.

Many previous studies have examined the effects of a single dose of UCA on various disease outcomes. However, a single UV exposure can increase skin levels of UCA in humans for weeks [20]. Here, we demonstrate neither prolonged dermal nor transient systemic increases in *cis*-UCA are able to protect against EAE.

To increase *cis* content via isomerization, we administered the *trans* isomer topically prior to UVB exposure. However, the conversion to the *cis* isomer was not any greater than in animals exposed to UVB alone. This is likely owing to the achievement of the photostationary state where no further conversion to *cis* will occur [21]. Despite reaching this level, protection was actually dampened when UVB was accompanied by topical application of the *trans* isomer.

C*is*-UCA levels in blood are low, and we were unable to detect significant differences in this measure between control and UVB-exposed mice. A single i.p. injection of UVB-exposed UCA caused a rapid rise in blood *cis*-UCA levels that returned to baseline within 4 h. These low and transient blood levels are likely explained by the presence of urocanase in the liver, which specifically metabolizes *cis*-UCA, and the high water solubility of the compound, causing it to be readily excreted.

It is clear that ultraviolet B exposure efficiently prevents EAE, though we find no evidence to support UCA isomerization as the mediator of this suppression. This is not the first study to discount UCA as a mediator of UV immunosuppression, or that UCA administration does not mimic the effects of UV exposure [17]. This lack of protection may not be surprising. Recent evidence has emerged demonstrating the essential role for regulatory B cells in the protection by UVB from EAE [22]. By contrast, urocanic acid appears to cause immunosuppression primarily via a T cell dependent manner [23, 24]. It is possible that the trend for lower vitamin D and *cis*-UCA levels in MS patients are each indicative of lower UV exposure, rather than as mediators of disease themselves. Additional work is needed to explore avenues other than vitamin D and urocanic acid to determine by what mechanism UVB is providing protection against autoimmune disease.

## Abbreviations

MS: Multiple sclerosis
25-OH-D: 25-hydroxyvitamin D
EAE: experimental autoimmune encephalomyelitis
UV(B): ultraviolet (B)
UCA: urocanic acid
